# Neoadjuvant ADT for Asian Patients Undergoing Robotic Radical Prostatectomy Is the Conversation Over?—A Propensity-Matched Comparison

**DOI:** 10.3390/cancers18040661

**Published:** 2026-02-18

**Authors:** John Joson Ng, Sean Lim, Alvin Lee, Yu Guang Tan, Kae Jack Tay, Henry Ho, John Yuen, Kenneth Chen

**Affiliations:** 1 Duke-NUS Medical School, Singapore 169857, Singapore; johnjoson.ng@u.duke.nus.edu; 2 Department of Urology, Singapore General Hospital, Singapore 169608, Singapore

**Keywords:** prostate cancer, neoadjuvant, surgery, radical prostatectomy, ADT

## Abstract

Background: Evidence for neoadjuvant androgen deprivation therapy (ADT) before radical prostatectomy (RP) remains inconclusive, and current guidelines do not endorse its routine use. Objective: We aimed to evaluate the impact of neoadjuvant ADT on surgical and oncologic outcomes in a Singaporean cohort undergoing radical prostatectomy. Design, setting, and participants: In this retrospective study, 1091 men underwent RP between 2013 and 2024; a total of 105 received neoadjuvant ADT and 986 did not. A 1:1 propensity score-matched analysis was performed on age, PSA, PSA density, Gleason score, clinical T-stage, and receipt of adjuvant therapies, yielding 105 matched pairs. Outcome measurements and statistical analysis: The primary outcome was biochemical recurrence (BCR). Secondary surgical outcomes included operative time, estimated blood loss, length of stay, catheter duration, and postoperative complications. Secondary oncologic outcomes included extracapsular extension, margin status, seminal vesicle invasion, lymph node involvement, clinical-to-pathological T-stage downstaging, Gleason score decrease, and PSA decrease. Kaplan–Meier survival and univariable linear and logistic regression analyses were used. Subgroup analysis was performed using stratified odds ratios to identify clinical subgroups that derived the greatest benefit from neoadjuvant ADT in terms of biochemical recurrence reduction. Results and limitations: After matching, neoadjuvant ADT was associated with a lower rate of extracapsular extension (30.8% vs. 51.4%, *p* = 0.004), positive surgical margins (18.4% vs. 39.4%, *p* = 0.002), lymph node involvement (1.0% vs. 13.0%, *p* = 0.002), and biochemical recurrence (4.8% vs. 18.1%, *p* = 0.005). There were no significant differences in operative time, blood loss, length of stay, or complication rates. Before matching, 2-year biochemical recurrence-free survival (BCR-FS) did not differ significantly (93.0% vs. 88.2%, log-rank *p* = 0.26), but after matching, BCR-FS favored ADT (93.0% vs. 81.8%, log-rank *p* = 0.02). Subgroup analysis showed that the reduction in biochemical recurrence with neoadjuvant ADT was more pronounced in patients with PSA density ≥ 0.20 ng/mL^2^, Gleason score ≥ 8, and clinical T3 disease. Limitations include the retrospective design and potential residual confounding. Conclusions: Neoadjuvant ADT prior to RP significantly reduces locoregional spread and biochemical recurrence without increasing perioperative morbidity. Prospective trials are needed to confirm its benefit in high-risk prostate cancer.

## Simple Summary

This study demonstrates that neoadjuvant ADT prior to radical prostatectomy significantly reduces biochemical recurrence, lymph node involvement, and positive surgical margins in a high-risk prostate cancer population. Importantly, these oncologic benefits are achieved without increasing perioperative complication rates or surgical morbidity. By using a propensity score-matched Asian cohort, the study adds region-specific evidence supporting the reconsideration of neoadjuvant ADT use. The findings highlight patient subgroups (e.g., PSA density ≥ 0.2 ng/mL^2^, Gleason ≥ 8, cT3) that may derive greater benefit from such intervention. Patient summary: We examined whether hormone therapy given before surgery helps men with prostate cancer. Our findings suggest that it reduces cancer spread and the chance of recurrence without increasing surgical risks. These benefits were especially clear in patients with high-risk disease.

## 1. Introduction

Prostate cancer (PCa) remains one of the most commonly diagnosed malignancies worldwide, with high-risk and very high-risk disease carrying substantial risk for recurrence and cancer-specific mortality despite definitive therapy [1,2]. In Singapore, PCa is the cancer of highest incidence in males, comprising 17.4% of all diagnosed cancers in 2018–2022 [3]. Contemporary registry data from Singapore have further highlighted the substantial morbidity burden associated with advanced and metastatic disease in the local population, underscoring the ongoing clinical challenge of achieving durable disease control [4]. Radical prostatectomy (RP), including robot-assisted approaches (RARP), is frequently used in multimodal treatment for localized and locally advanced PCa. However, achieving durable oncological control in PCa remains a significant challenge, with 20–50% of men with PCa developing biochemical recurrence (BCR) within 10 years after initial definitive therapy [5,6].

Neoadjuvant androgen deprivation therapy (ADT)—typically consisting of gonadotropin-releasing hormone (GnRH) agonists or antagonists such as leuprorelin, goserelin, triptorelin, or degarelix—has been explored in this context with the goal of reducing tumor burden, facilitating surgical resection, and improving pathological outcomes [7]. Prior studies and meta-analyses have demonstrated reductions in pathological staging [8], extracapsular extension [8,9,10], lymph node involvement [8,10,11], and positive surgical margin (PSM) [7,8,9,10] rates following neoadjuvant ADT. However, its ability to improve long-term oncological outcomes such as BCR, metastasis-free survival, or overall survival remains controversial [7,8,10]. Reported discrepancies across studies likely reflect substantial methodological heterogeneity, including differences in study era, surgical technique, patient risk profiles, duration and type of neoadjuvant therapy, and analytical approaches. Many early trials were conducted prior to the widespread adoption of robot-assisted radical prostatectomy and relied on less sensitive prostate-specific antigen assays and variable definitions of biochemical recurrence, limiting comparability across cohorts. In addition, incomplete adjustment for downstream treatments such as adjuvant radiotherapy or postoperative ADT may confound attribution of oncological benefit to neoadjuvant therapy alone. Importantly, variation in statistical modeling strategies and risk stratification frameworks further contributes to heterogeneity in reported outcomes. As highlighted in recent work emphasizing the prognostic value of survival modeling in prostate cancer and the growing role of data science in individualized oncology care, robust analytical design and transparent modeling are increasingly critical for interpreting real-world outcomes and guiding personalized treatment decisions [12,13]. These considerations underscore the need for contemporary, methodologically rigorous analyses evaluating neoadjuvant ADT in modern clinical practice. As a result, current international guidelines do not recommend NHT prior to surgery outside of clinical trials [14,15,16,17].

While recent trials have explored neoadjuvant strategies incorporating androgen receptor signaling inhibitors (ARSIs) [18], these regimens remain investigational and are not routinely used in most clinical settings. In contrast, conventional ADT with GnRH agonists or antagonists remains widely employed, particularly in contexts where ARSIs are not yet standard or where treatment delays—such as those seen during the COVID-19 pandemic—necessitate bridging therapy [19,20]. However, high-quality evidence evaluating the real-world impact of standard neoadjuvant ADT on long-term oncological outcomes remains limited, especially in Asian populations [21]. In the context of ongoing clinical uncertainty and the absence of consensus recommendations supporting routine neoadjuvant ADT prior to radical prostatectomy, prospective randomized trials remain limited and logistically challenging, particularly for evaluating long-term oncological endpoints. In this setting, retrospective real-world analyses provide an important initial approach to assess treatment patterns and outcomes in contemporary clinical practice, generate hypothesis-driven insights, and inform the design of future prospective studies.

In this retrospective study, we evaluated the effect of neoadjuvant ADT on oncological and surgical outcomes in a Singaporean cohort of patients undergoing radical prostatectomy, with the aim of addressing the limited real-world evidence from Asian populations. Using propensity score matching (PSM), we aimed to minimize selection bias and investigate whether neoadjuvant ADT was associated with improved BCR-free survival or pathological outcomes. We also examined whether certain clinical subgroups—defined by PSA density, Gleason score, and T stage—derive greater benefit from preoperative hormonal therapy. This study adds to the growing body of real-world data by addressing an underrepresented regional evidence gap regarding neoadjuvant ADT use in prostate cancer. We hypothesized that neoadjuvant ADT prior to radical prostatectomy is associated with improved biochemical recurrence-free survival, with exploratory evaluation of pathological and surgical outcomes.

## 2. Patients and Methods

### 2.1. Study Design and Population

This was a retrospective cohort study conducted at Singapore General Hospital. Singapore General Hospital is the largest tertiary academic medical centre in Singapore and a national referral centre for urological oncology, including prostate cancer. Patients diagnosed with clinically localized or locally advanced prostate cancer who underwent radical prostatectomy between 1 January 2013 and 30 April 2024 were included in this study. A total of 1091 patients underwent radical prostatectomy during the study period, of whom 105 received neoadjuvant androgen deprivation therapy and 986 underwent surgery without neoadjuvant ADT. Patients who received neoadjuvant ADT with leuprorelin, goserelin, triptorelin, or degarelix prior to surgery were categorized into the treatment group. Among patients receiving neoadjuvant ADT, the most commonly used agent was leuprolide (*n* = 72), followed by goserelin (*n* = 19), degarelix (*n* = 13), and triptorelin (*n* = 1). The treatment group was not filtered for duration of neoadjuvant ADT before their operation. Patients who underwent surgery without any neoadjuvant ADT formed the control group.

### 2.2. Data Collection

Baseline demographic and clinical variables, including age at diagnosis, PSA at diagnosis, PSA density, Gleason score group, and clinical T stage, were extracted from electronic medical records. Perioperative variables (including operative time, estimated blood loss, length of stay, catheter duration), postoperative complications (such as anastomotic leak, wound infection, and other adverse events), and pathological features (extracapsular extension, seminal vesicle invasion, margin status, lymph node involvement, and pathological downstaging) were similarly collected. These variables were documented as part of routine clinical care and were later designated a priori as secondary outcomes for this analysis. Follow-up PSA measurements were used to determine biochemical recurrence, defined as a postoperative PSA ≥ 0.2 ng/mL on two consecutive measurements were used to determine biochemical recurrence.

### 2.3. Propensity Score Matching

To reduce selection bias and confounding, patients in the neoadjuvant ADT group were matched 1:1 to controls using propensity score matching. Four matching algorithms were explored: nearest neighbor, nearest neighbor with a caliper of 0.2, full matching, and optimal matching. Each approach was evaluated using covariate balance diagnostics, including standardized mean differences and visual inspection via Love plots. Optimal matching was ultimately selected, as it achieved superior covariate balance across all matching variables without sacrificing sample size. Matching variables included age, PSA, PSA density, Gleason score group, clinical T stage, and receipt of adjuvant therapy. This approach enabled more precise estimation of treatment effects while minimizing residual confounding.

### 2.4. Outcomes

The primary endpoint was biochemical recurrence. Biochemical recurrence-free survival (BCR-FS), defined as the time from surgery to the date of confirmed biochemical recurrence or last follow-up, was analyzed separately using Kaplan–Meier methods.

Secondary outcomes included both surgical and oncological endpoints. Surgical outcomes comprised total operative time, estimated blood loss, length of stay, and catheter duration, each reported as median with interquartile range. In addition, postoperative complications were evaluated and included events such as anastomotic leak, deep vein thrombosis, myocardial infarction, pneumonia, postoperative blood transfusion, prolonged ileus, pulmonary embolism, rectal injury, urinary retention, reoperation, wound infection, and other complications.

Oncological outcomes included the presence of locoregional spread, defined by extracapsular extension, margin involvement, seminal vesicle invasion, and lymph node involvement. Tumor downstaging metrics were also assessed, including clinical-to-pathological T-stage downstaging, Gleason score decrease, and Gleason group downstage. PSA response was evaluated through PSA decrease and PSA group downstaging at 3 months postoperatively.

### 2.5. Statistical Analysis

Descriptive statistics were used to summarize patient characteristics. Before matching, comparisons between groups were performed using chi-square or Fisher’s exact tests for categorical variables, and *t*-tests or Mann–Whitney U tests for continuous variables. After propensity score matching, paired analyses were conducted: McNemar’s or exact McNemar’s tests for categorical variables, and paired *t*-tests or Wilcoxon signed-rank tests for continuous variables.

Biochemical recurrence-free survival was estimated using Kaplan–Meier methods, and differences between groups were assessed using the log-rank test.

Univariable linear regression was used to compare continuous perioperative outcomes—including operative time, estimated blood loss, length of stay, and catheter duration—between treatment groups. Beta coefficients with corresponding 95% confidence intervals and *p*-values were reported.

Univariable logistic regression was used to evaluate the association between neoadjuvant androgen deprivation therapy (ADT) and binary or categorical outcomes, including pathological features and biochemical recurrence. Separate models were fitted for each outcome variable, both before and after matching. Odds ratios (ORs) with 95% confidence intervals were reported, with OR < 1 indicating that the outcome was less likely in the neoadjuvant ADT group.

The primary endpoint of this study was biochemical recurrence. Analyses of secondary surgical, pathological, and subgroup outcomes were conducted in an exploratory, hypothesis-generating manner. Given the limited number of biochemical recurrence events, formal statistical interaction testing for subgroup effects was not performed. Accordingly, no formal adjustment for multiple comparisons was applied, and results are interpreted cautiously with emphasis on effect sizes and confidence intervals rather than *p*-values alone.

A two-sided *p*-value < 0.05 was considered statistically significant. Statistical analysis was performed using R version [4.5.0].

## 3. Results

### 3.1. Baseline Characteristics

In the unmatched cohort, patients who received neoadjuvant ADT (*n* = 105) were older (*p* = 0.027) and had higher PSA (*p* < 0.001), higher baseline PSA density (*p* = 0.003), higher max PI-RADS score (*p* < 0.001), higher Gleason score groups (*p* < 0.001), and more advanced clinical T stages (*p* < 0.001) compared to those who did not (*n* = 986) (Table 1A). There were also differences in receipt of adjuvant RT and adjuvant ADT, and median follow-up period between the two groups. 1:1 propensity score matching on age, PSA, PSA density, Gleason group, clinical T stage, and receipt of adjuvant therapy, 105 matched pairs were generated with well-balanced covariates (all absolute standardized mean differences [ASMD] < 0.1) (Figure S1). Optimal matching was ultimately selected as all covariates had ASMD ≤ 0.05. The baseline characteristics of the matched cohort is presented in Table 1.

### 3.2. Surgical and Perioperative Outcomes

Following 1:1 propensity score matching, there were no statistically significant differences in operative time, estimated blood loss, or length of stay between patients who received neoadjuvant ADT and those who did not (Table 1B and Table 2B).

**Figure 1:**
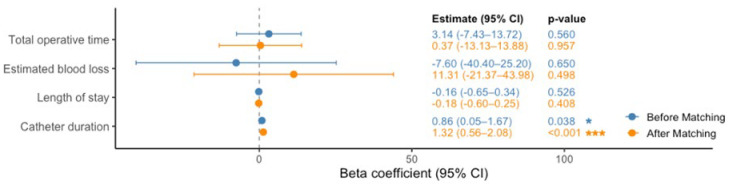
Effect of Neoadjuvant ADT on Surgical Outcomes: Univariable Linear Regression Before and After Matching. Forest plot of beta coefficients (95% CI) for the association between neoadjuvant ADT and surgical outcomes (continuous variables), shown before (blue) and after (orange) 1:1 propensity-score matching. Outcomes include total operative time (minutes), estimated blood loss (mL), length of stay (days), and catheter duration (days). Point estimates are plotted with horizontal bars indicating 95% confidence intervals; corresponding numeric estimates and *p*-values are listed to the right. Asterisks denote significance levels: * *p* < 0.05, *** *p* < 0.001.

**Figure 2:**
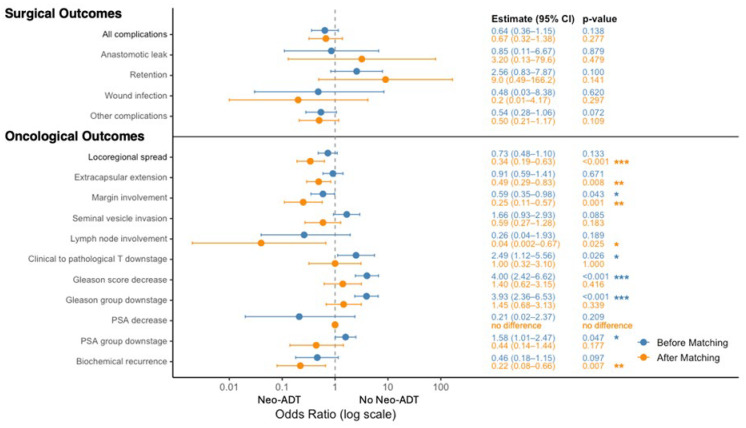
Impact of Neoadjuvant ADT on Surgical and Oncological Outcomes: Odds Ratios Pre- and Post-Matching. Forest plot of odds ratios (ORs) for the association between neoadjuvant androgen deprivation therapy (ADT) and key surgical and oncological outcomes, shown on a logarithmic scale before (blue) and after (orange) 1:1 propensity-score matching. Surgical outcomes (**top**): all complications, anastomotic leak, urinary retention, wound infection, and other complications. Oncological outcomes (**bottom**): locoregional spread, extracapsular extension, positive surgical margins, seminal vesicle invasion, lymph node involvement, clinical-to-pathological T downstaging, Gleason score decrease, Gleason group downstaging, PSA decrease (no adjusted OR available), PSA group downstaging, and biochemical recurrence. Each marker denotes the point estimate of the OR, with horizontal lines indicating the 95% confidence interval. Corresponding numeric estimates and *p*-values are listed to the right of each row. Asterisks denote significance levels: * *p* < 0.05, ** *p* < 0.01, *** *p* < 0.001.

However, univariate linear regression analyses revealed that neoadjuvant ADT was associated with a significantly longer catheter duration both before matching (β = 0.86 days, 95% CI [0.05, 1.67], *p* = 0.038) and after matching (β = 1.32 days, 95% CI [0.11, 2.47], *p* < 0.001) (Figure 1). Univariate logistic regression analyses revealed comparable likelihood of complications (Figure 2). This suggests a modest but statistically significant increase in catheterisation time associated with neoadjuvant therapy, while other surgical outcomes remained comparable.

### 3.3. Oncological Outcomes

After matching, patients who received neoadjuvant ADT exhibited lower rates of several adverse pathological features, including locoregional spread, encapsulating extracapsular extension, margin involvement, and lymph node involvement (Table 1C and Table 2C). Seminal vesicle invasion, specifically, was not significantly different before and after matching.

In univariate logistic regression analyses (Figure 2), neoadjuvant ADT was significantly associated with reduced odds of extracapsular extension (OR = 0.49, 95% CI [0.29–0.83], *p* = 0.008), positive surgical margins (OR = 0.25, 95% CI [0.11–0.57], *p* = 0.001), lymph node involvement (OR = 0.04, 95% CI [0.002–0.67], *p* = 0.025), and biochemical recurrence (OR = 0.22, 95% CI [0.08–0.66], *p* = 0.007). There were also non-significant trends suggesting lower risk of seminal vesicle invasion (OR = 0.59, *p* = 0.183). Several apparent indicators of pathological and biochemical downstaging—including clinical-to-pathological T downstaging, Gleason score and group reduction, postoperative PSA decrease, and PSA group downstaging—were statistically significant in the unmatched cohort. However, these associations diminished in magnitude and lost statistical significance after propensity score matching, suggesting that the initial differences were likely confounded by baseline risk imbalances.

### 3.4. Biochemical Recurrence

**Figure 3:**
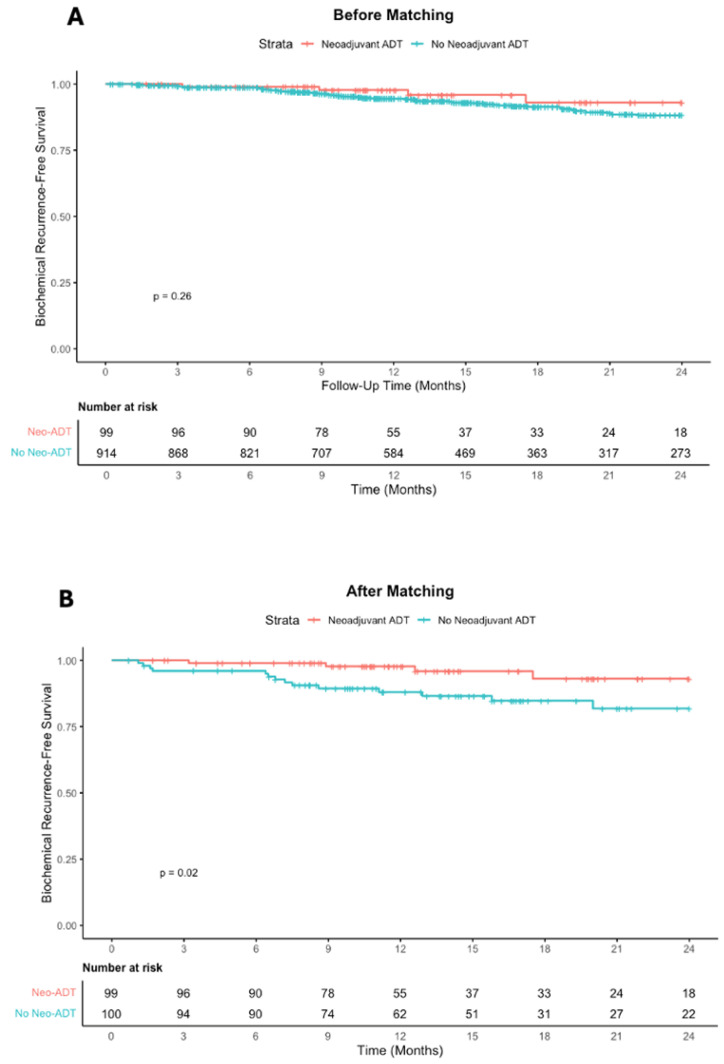
Biochemical Recurrence-Free Survival With and Without Neoadjuvant ADT. Kaplan–Meier analysis of biochemical recurrence-free survival comparing patients who received neoadjuvant androgen deprivation therapy (Neo-ADT; red) versus those who did not (No Neo-ADT; blue) (**A**) before and (**B**) after 1:1 propensity score matching. Tick marks indicate censored observations. Below each plot are the numbers at risk at 0 to 24 months, in three month intervals. *p*-value was calculated by log-rank analysis.

After matching, at a median follow-up of 14 months overall, biochemical recurrence occurred less frequently in the Neo-ADT group than in controls (4.8% vs. 18.1%, *p* = 0.005) (Table 2). Patients who received neoadjuvant ADT were less likely to experience biochemical recurrence overall (OR: 0.22, 95% CI: 0.08–0.66, *p* = 0.007). Kaplan–Meier analysis showed a significant benefit in 2-year biochemical recurrence-free survival for those receiving neoadjuvant ADT (93.0% vs. 81.8%, log-rank *p* = 0.02) after matching, whereas the difference was not significant before matching (log-rank *p* = 0.26) (Figure 3).

Although Kaplan–Meier estimates can be generated with small sample sizes, reliable interpretation typically requires an adequate number of events within each subgroup. In the present study, biochemical recurrence events within individual ADT-agent strata were sparse, resulting in statistically unstable survival estimates. Accordingly, agent-specific Kaplan–Meier curves were treated as exploratory and are presented in the Supplementary Materials (Supplementary Figure S2) for descriptive purposes only.

In a pre-specified parsimonious Cox proportional hazards sensitivity analysis, neoadjuvant ADT remained independently associated with improved biochemical recurrence-free survival (HR 0.28, 95% CI 0.09–0.80; *p* = 0.018; Supplementary Table S1).

### 3.5. Subgroup Analysis

**Figure 4:**
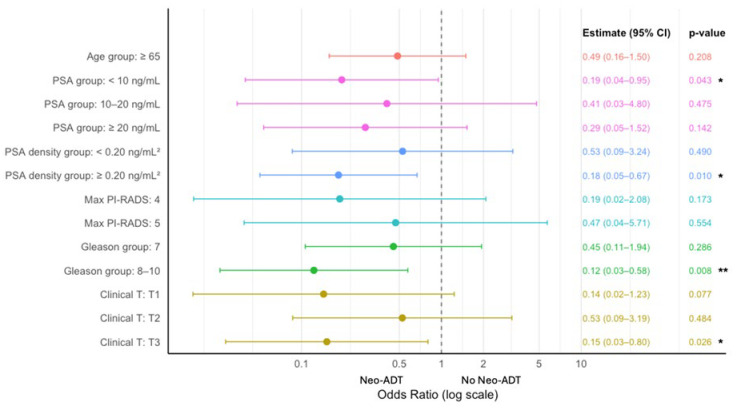
Subgroup Analysis of Biochemical Recurrence Risk: Stratified Odds Ratios for Neo-ADT vs. No Neo-ADT. Forest plot of stratum-specific odds ratios (ORs) and 95% confidence intervals for biochemical recurrence comparing neoadjuvant ADT versus no ADT across baseline patient subgroups. Each horizontal line represents one subgroup—age (≥65 years), PSA level (<10, 10–20, ≥20 ng/mL), PSA density (<0.20 vs. ≥0.20 ng/mL^2^), maximum PI-RADS score (4 vs. 5), Gleason group (7 vs. 8–10), and clinical T stage (T1, T2, T3)—with the point estimate plotted on a logarithmic *x*-axis. A vertical line at OR = 1 denotes no difference in recurrence risk. Subgroups with ORs < 1 favor benefit from neoadjuvant ADT. The numeric ORs, 95% CIs, and *p*-values are shown to the right of each row; statistically significant associations (*p* < 0.05) indicate which patient groups derive the greatest reduction in biochemical recurrence with neoadjuvant ADT. Asterisks denote significance levels: * *p* < 0.05, ** *p* < 0.01.

Forest plot analysis of stratum-specific odds ratios showed that the reduction in biochemical recurrence with neoadjuvant ADT was more pronounced in patients with a PSA < 10 ng/mL, a PSA density ≥ 0.20 ng/mL [2], a Gleason score ≥ 8, and clinical T3 disease. These associations were statistically significant (*p* < 0.05), suggesting that patients with high-risk features may derive greater benefit from neoadjuvant ADT (Figure 4).

Although additional subgroup stratification was considered, the limited number of events within individual subgroups after propensity score matching precluded further reliable analysis, and such results would have been statistically unstable.

## 4. Discussion

Our findings provide new real-world evidence supporting the use of neoadjuvant androgen deprivation therapy (ADT) prior to radical prostatectomy (RP) in patients with high-risk prostate cancer. In this propensity score-matched cohort from Singapore, neoadjuvant ADT was associated with significant reductions in extracapsular extension, PSM, lymph node involvement, and biochemical recurrence. Importantly, these oncologic benefits did not come at the expense of increased operative time, blood loss, hospital stay, or perioperative complications.

Our results are consistent with several prior meta-analyses and retrospective studies demonstrating reductions in locoregional spread and improvements in pathological outcomes with neoadjuvant ADT [7,9,10]. These findings echo recent cohort studies from Asia, such as Nezasa et al. [21], that support the oncologic utility of neoadjuvant ADT in high-risk disease. Notably, our matched analysis showed a marked improvement in 2-year biochemical recurrence-free survival (BCR-FS), suggesting that neoadjuvant therapy may offer durable oncological benefit in appropriately selected patients.

Nonetheless, the clinical value of neoadjuvant ADT remains controversial. Randomized trials such as those by Soloway et al. [22] and those reviewed by Shelley et al. [10] demonstrated improvements in pathological outcomes but did not show survival advantages, which has contributed to the current hesitancy in clinical guidelines. Historical concerns regarding increased periprostatic fibrosis [23] and distorted anatomical planes [24]—potentially complicating nerve-sparing surgery—have further tempered enthusiasm for neoadjuvant ADT. However, emerging evidence, including our own findings, suggests that such concerns may be less relevant in the modern era of robot-assisted surgery (RARP), where operative time, blood loss, and complication rates remain unaffected by prior ADT exposure [25,26].

A key strength of our study lies in its accounting for adjuvant therapy status during propensity score matching. By balancing for receipt of postoperative ADT or radiotherapy, we were able to more accurately isolate the effect of neoadjuvant intervention. Many earlier studies did not account for this potential confounder, complicating the attribution of outcomes [27]. Our design enhances internal validity and provides more credible evidence that the improvements observed are due to neoadjuvant ADT itself.

Our results also highlight the relevance of neoadjuvant ADT as a bridging strategy—particularly during the COVID-19 pandemic, when multiple urological societies endorsed ADT to delay definitive surgery without compromising oncologic outcomes [19,20,28]. This application remains pertinent in resource-limited environments or in patients awaiting surgical slots. Our findings offer reassurance that bridging ADT may not only be oncologically safe, but may even confer benefit in patients with biologically aggressive tumors.

Previous studies have consistently identified high pre-treatment PSA density and a Gleason score ≥ 8 as independent predictors of biochemical recurrence following radical prostatectomy. For instance, PSA density has been shown to correlate with early treatment failure and adverse pathological features [29]. Similarly, multiple studies have reported that tumors with Gleason score ≥ 8 are associated with significantly higher recurrence rates and poorer long-term outcomes after surgery alone [30,31,32]. Notably, in our stratified analysis, these very subgroups—patients with PSA density ≥ 0.20 ng/mL^2^ and Gleason score ≥ 8—demonstrated the greatest benefit from neoadjuvant ADT, with significantly lower rates of biochemical recurrence compared to controls. This suggests that neoadjuvant therapy may be particularly effective in patients with biologically aggressive disease, potentially offsetting the elevated recurrence risk conferred by unfavorable baseline characteristics.

Our findings further reinforce the concept that tumor burden and aggressiveness—not just clinical stage—may influence response to hormonal priming. In addition to Gleason score and PSA density, we observed that patients with clinical T3 disease also derived substantial benefit from neoadjuvant therapy. Interestingly, we also observed a significant benefit in patients with PSA < 10 ng/mL (OR 0.19, 95% CI 0.04–0.95), while those with PSA ≥ 20 ng/mL did not show a statistically significant reduction in recurrence. This finding challenges the assumption that only high-PSA patients derive benefit from neoadjuvant therapy and suggests that certain low-PSA tumors may be more biologically responsive to hormonal modulation. These insights support a tailored, risk-adapted approach to patient selection for neoadjuvant ADT. While other studies have also explored predictive factors for neoadjuvant benefit—such as Wang et al. [33], who reported poorer responses in tumors with cribriform histology, and Powell et al. [34], who noted improved downstaging in cT3 disease—few have incorporated PSA density into their risk stratification frameworks [29,35]. Our application of PSA density, a clinically accessible but underutilized parameter, offers a novel and pragmatic tool for optimizing patient selection in future neoadjuvant strategies.

Additionally, we observed that certain oncologic variables—such as Gleason score reduction, Gleason group downstaging, PSA response, and PSA group downstaging—were significantly more common in patients receiving neoadjuvant ADT prior to matching. However, after adjustment, these differences were no longer significant. This suggests that the apparent downstaging effects may have been confounded by baseline risk factors: patients selected for ADT were older and had higher PSA, PSA density, Gleason grade, clinical T stage, and PI-RADS scores. Once these differences were accounted for, the magnitude of downstaging diminished, underscoring the importance of addressing confounding in retrospective analyses. Our matched study design mitigates this risk and allows for more accurate estimation of neoadjuvant effects.

This study has several limitations. First, its retrospective design is inherently susceptible to bias and confounding, despite robust propensity score matching and sensitivity analyses; residual confounding therefore cannot be fully excluded. Second, the relatively short median follow-up duration may limit the detection of late recurrences or long-term survival outcomes. Third, the sample size of the neoadjuvant group remains modest, and treatment heterogeneity (in agent type and duration) could influence outcomes. Fourth, the lack of central pathology review may introduce variability in grading and staging assessments. Although clinical T stage was available for most patients, preoperative prostate MRI was not uniformly performed across the study period, reflecting the retrospective, real-world nature of the cohort rather than systematically low MRI utilization, and may have introduced variability in baseline staging accuracy. Additionally, surgical era was not explicitly adjusted for in the analysis, and temporal improvements in robotic technique, surgeon experience, and extent of lymph node dissection may therefore represent unmeasured confounders influencing operative and oncologic outcomes. Finally, given the limited number of biochemical recurrence events, formal statistical interaction testing for subgroup effects was not feasible; accordingly, secondary and subgroup analyses were exploratory, and findings should be interpreted as hypothesis-generating rather than confirmatory.

Despite these limitations, our study provides strong evidence that neoadjuvant ADT is both oncologically effective and surgically safe. These findings support its selective use in patients with high-risk disease and justify further prospective trials to confirm these benefits. Such trials should aim to stratify patients by clinical risk factors, treatment duration, and integration of next-generation androgen receptor inhibitors to optimize outcomes.

## 5. Conclusions

Neoadjuvant androgen deprivation therapy prior to radical prostatectomy was associated with reduced locoregional tumor spread and biochemical recurrence, without adversely affecting surgical morbidity or perioperative outcomes. These findings suggest a role for neoadjuvant ADT in selected high-risk prostate cancer patients, particularly those with clinical T3 disease, a PSA density ≥ 0.20 ng/mL^2^, or a Gleason score ≥8. Findings should be interpreted cautiously given the retrospective design. Further prospective, randomized trials are warranted to validate these benefits and establish standardized treatment protocols.

## Figures and Tables

**Table 1:** Baseline Characteristics, Surgical Outcomes, and Oncological Outcomes of Patients With and Without Neoadjuvant Androgen Deprivation Therapy (Unmatched Cohort). Comparison of patients undergoing robotic radical prostatectomy with and without neoadjuvant androgen deprivation therapy (ADT) in the unmatched cohort: (**A**) Baseline demographic, clinical, and pathological characteristics, including age group, prostate-specific antigen (PSA) level and density, maximum Prostate Imaging–Reporting and Data System (PI-RADS) score, Gleason group, clinical T stage, use of adjuvant radiotherapy (RT), use of adjuvant ADT, and missing data indicators. (**B**) Perioperative and surgical outcomes. (**C**) Oncological outcomes.

Characteristic	Level	Neoadjuvant ADT	No Neoadjuvant ADT	*p*-Value	SMD	
(**A**) **Baseline demographic, clinical, and pathological characteristics**						
n		105	986			
Age (Median [Q1, Q3])		67.5 [63, 71.5]	66 [62, 70]	<0.003		**
Age group	<65	31 (29.5)	408 (41.4)	0.027	0.264	*
	≥65	73 (69.5)	576 (58.4)			
	Missing	1 (1.0)	2 (0.2)			
PSA at diagnosis (Median [Q1, Q3])		10.3 [6.43, 19.28]	8 [5.91, 11.97]	<0.001		***
PSA group	<10 ng/mL	49 (46.7)	625 (63.4)	<0.001	0.446	***
	10–20 ng/mL	28 (26.7)	253 (25.7)			
	≥20 ng/mL	25 (23.8)	88 (8.9)			
	Missing	3 (2.9)	20 (2.0)			
PSA density (Median [Q1, Q3])		0.282 [0.222, 0.491]	0.238 [0.159, 0.381]	<0.001		***
PSA density group	<0.20 ng/mL^2^	20 (19.0)	350 (35.5)	0.003	0.379	**
	≥0.20 ng/mL^2^	73 (69.5)	534 (54.2)			
	Missing	12 (11.4)	102 (10.3)			
Max PI-RADS score	PI-RADS 2	0 (0.0)	2 (0.2)	<0.001	0.525	***
	PI-RADS 3	1 (1.0)	13 (1.3)			
	PI-RADS 4	17 (16.2)	82 (8.3)			
	PI-RADS 5	18 (17.1)	44 (4.5)			
	Missing	69 (65.7)	845 (85.7)			
Gleason group	≤6	6 (5.7)	194 (19.7)	<0.001	0.814	***
	7	46 (43.8)	626 (63.5)			
	8–10	51 (48.6)	153 (15.5)			
	Missing	2 (1.9)	13 (1.3)			
Clinical T	T1	59 (56.2)	644 (65.3)	<0.001	0.361	***
	T2	22 (21.0)	246 (24.9)			
	T3	24 (22.9)	96 (9.7)			
Adjuvant RT	Did not receive	99 (94.3)	752 (76.3)	<0.001	0.533	***
	Received	1 (1.0)	16 (1.6)			
	Missing	5 (4.8)	218 (22.1)			
Adjuvant ADT	Did not receive	80 (76.2)	710 (72.0)	<0.001	0.611	***
	Received	19 (18.1)	53 (5.4)			
	Missing	6 (5.7)	223 (22.6)			
Follow-up period, months (Median [Q1, Q3])		13.4 [9.6, 21.2]	16.1 [10.4, 31.2]	0.014	0.21	*
(**B**) **Perioperative and surgical outcomes**						
Total operative time, min (Median [IQR])		235.0 [210.0, 270.0]	235.0 [205.0, 275.0]	0.569	0.062	
Estimated blood loss, mL (Median [IQR])		200.0 [100.0, 200.0]	200.0 [100.0, 250.0]	0.981	0.054	
Length of stay, days (Median [IQR])		2.0 [2.0, 3.0]	2.0 [2.0, 3.0]	0.433	0.078	
Catheter duration, days (Median [IQR])		8.0 [7.0, 9.5]	8.0 [7.0, 8.0]	0.057	0.226	
All complications		14 (13.6)	187 (19.7)	0.173	0.164	
Anastomotic leak		1 (1.0)	11 (1.1)	1.000	0.016	
Deep vein thrombosis		0 (0.0)	2 (0.2)	1.000	0.064	
Pneumonia		0 (0.0)	1 (0.1)	1.000	0.045	
Post-op blood transfusion		0 (0.0)	7 (0.7)	0.821	0.122	
Prolonged ileus		0 (0.0)	3 (0.3)	1.000	0.078	
Pulmonary embolism		0 (0.0)	1 (0.1)	1.000	0.045	
Rectal injury		0 (0.0)	1 (0.1)	1.000	0.045	
Retention		4 (3.8)	15 (1.5)	0.190	0.142	
Wound infection		0 (0.0)	9 (0.9)	0.674	0.136	
Other complications		10 (9.5)	161 (16.3)	0.093	0.204	
(**C**) **Oncological Outcomes**						
Locoregional spread		41 (39.0)	461 (46.8)	0.161	0.156	
Extracapsular extension		32 (30.8)	322 (32.8)	0.753	0.044	
Margin involvement		19 (18.4)	269 (27.8)	0.055	0.224	
Seminal vesicle invasion		16 (15.4)	97 (9.9)	0.116	0.166	
Lymph node involvement		1 (1.0)	33 (3.7)	0.261	0.180	
Clinical to pathological T downstage		8 (7.8)	32 (3.3)	0.043	0.198	*
Gleason score decrease		28 (36.8)	121 (12.7)	<0.001	0.582	***
Gleason group downstage		27 (35.5)	117 (12.3)	<0.001	0.566	***
PSA decrease at 3-months post-op		90 (98.9)	845 (99.8)	0.683	0.106	
PSA group downstage at 3-months post-op		40 (47.1)	291 (36.1)	0.060	0.225	
Biochemical recurrence		5 (4.8)	97 (9.8)	0.128	0.196	

Continuous variables are presented as the median with interquartile range (Q1–Q3), and categorical variables are presented as numbers (percentages). *p*-values reflect between-group comparisons prior to matching and were calculated using χ^2^ or Fisher’s exact tests for categorical variables and appropriate non-parametric tests for continuous variables. Standardized mean differences (SMDs) are reported to quantify the magnitude of imbalance between groups. Asterisks denote significance levels: * *p* < 0.05, ** *p* < 0.01, *** *p* < 0.001. Sample sizes: *n* = 105 neoadjuvant ADT, *n* = 986 no neoadjuvant ADT. Abbreviations: ADT, androgen deprivation therapy; PI-RADS, Prostate Imaging–Reporting and Data System; PSA, prostate-specific antigen; RT, radiotherapy.

**Table 2:** Baseline Characteristics, Surgical Outcomes, and Oncological Outcomes of Patients With and Without Neoadjuvant Androgen Deprivation Therapy After Propensity Score Matching. Comparison of patients treated with neoadjuvant androgen deprivation therapy (ADT) and matched controls following propensity score matching: (**A**) Baseline demographic, clinical, and pathological characteristics, including age group, prostate-specific antigen (PSA) level and density, maximum Prostate Imaging–Reporting and Data System (PI-RADS) score, Gleason group, clinical T stage, use of adjuvant radiotherapy (RT), use of adjuvant ADT, and missing data indicators. (**B**) Perioperative and surgical outcomes. (**C**) Oncological outcomes.

Characteristic	Level	Neoadjuvant ADT	No Neoadjuvant ADT	*p*-Value	SMD	
(**A**) **Baseline demographic, clinical, and pathological characteristics**						
Locoregional spread		41 (39.0)	461 (46.8)	0.161	0.156	
Extracapsular extension		32 (30.8)	322 (32.8)	0.753	0.044	
Margin involvement		19 (18.4)	269 (27.8)	0.055	0.224	
Seminal vesicle invasion		16 (15.4)	97 (9.9)	0.116	0.166	
Lymph node involvement		1 (1.0)	33 (3.7)	0.261	0.180	
Clinical to pathological T downstage		8 (7.8)	32 (3.3)	0.043	0.198	*
Gleason score decrease		28 (36.8)	121 (12.7)	<0.001	0.582	***
Gleason group downstage		27 (35.5)	117 (12.3)	<0.001	0.566	***
PSA decrease at 3-months post-op		90 (98.9)	845 (99.8)	0.683	0.106	
PSA group downstage at 3-months post-op		40 (47.1)	291 (36.1)	0.060	0.225	
Biochemical recurrence		5 (4.8)	97 (9.8)	0.128	0.196	
(**B**) **Perioperative and surgical outcomes**						
Total operative time, min (Median [IQR])		235.0 [210.0, 270.0]	240.0 [205.0, 270.0]	0.870	0.012	
Estimated blood loss, mL (Median [IQR])		200.0 [100.0, 200.0]	200.0 [100.0, 200.0]	0.826	0.097	
Length of stay, days (Median [IQR])		2.0 [2.0, 3.0]	2.0 [2.0, 3.0]	0.428	0.116	
Catheter duration, days (Median [IQR])		8.0 [7.0, 9.5]	8.0 [7.0, 8.0]	0.076	0.456	
All complications	Yes	14 (13.6)	20 (19.4)	0.348	0.157	
Anastomotic leak	Yes	1 (1.0)	0 (0.0)	1.000	0.139	
Deep vein thrombosis	Yes	0 (0.0)	0 (0.0)	1	0	
Myocardial infarction	Yes	0 (0.0)	0 (0.0)	1	0	
Pneumonia	Yes	0 (0.0)	0 (0.0)	1	0	
Post-op blood transfusion	Yes	0 (0.0)	0 (0.0)	1	0	
Prolonged ileus	Yes	0 (0.0)	0 (0.0)	1	0	
Pulmonary embolism	Yes	0 (0.0)	0 (0.0)	1	0	
Rectal injury	Yes	0 (0.0)	0 (0.0)	1	0	
Retention	Yes	4 (3.8)	0 (0.0)	0.130	0.281	
Wound infection	Yes	0 (0.0)	2 (1.9)	0.469	0.199	
Other complications	Yes	10 (9.5)	18 (17.1)	0.155	0.226	
(**C**) **Oncological outcomes**						
Locoregional spread	Yes	41 (39.0)	68 (64.8)	<0.001	0.533	***
Extracapsular extension	Yes	32 (30.8)	54 (51.4)	0.004	0.429	**
Margin involvement	Yes	19 (18.4)	41 (39.4)	0.002	0.475	**
Seminal vesicle invasion	Yes	16 (15.4)	23 (21.9)	0.302	0.168	
Lymph node involvement	Yes	1 (1.0)	13 (13.0)	0.002	0.484	**
Clinical to pathological T downstage	Yes	8 (7.8)	8 (7.6)	1.000	0.006	
Gleason score decrease	Yes	28 (36.8)	31 (30.7)	0.485	0.130	
Gleason group downstage	Yes	27 (35.5)	29 (28.7)	0.423	0.146	
PSA decrease at 3-months post-op	Yes	90 (98.9)	90 (98.9)	1.000	<0.001	
PSA group downstage at 3-months post-op	Yes	40 (47.1)	44 (53.7)	0.485	0.132	
Biochemical recurrence	Yes	5 (4.8)	19 (18.1)	0.005	0.429	**

Continuous variables are presented as the median with interquartile range (Q1–Q3), and categorical variables are presented as numbers (percentages). *p*-values represent post-matching between-group comparisons and were calculated using χ^2^ or Fisher’s exact tests for categorical variables and appropriate non-parametric tests for continuous variables. Standardized mean differences (SMDs) demonstrate covariate balance after matching. Asterisks denote significance levels: * *p* < 0.05, ** *p* < 0.01, *** *p* < 0.001. Abbreviations: ADT, androgen deprivation therapy; PI-RADS, Prostate Imaging–Reporting and Data System; PSA, prostate-specific antigen; RT, radiotherapy.

## Data Availability

Data supporting the findings of this study are available from the corresponding author upon reasonable request.

## Supplementary Materials

The following supporting information can be downloaded at: https://www.mdpi.com/article/10.3390/cancers18040661/s1, Figure S1: Covariate Balance Across Matching Algorithms: Absolute Standardized Mean Differences Before and After Matching; Figure S2: Agent-specific Kaplan–Meier curves for biochemical recurrence-free survival comparing neoadjuvant androgen deprivation therapy (ADT) recipients with their matched control cohorts. (a) Leuprorelin acetate (b) Goserelin (c) Degarelix. Owing to the limited number of biochemical recurrence events within individual ADT agent subgroups, these analyses are underpowered for formal statistical comparison and are presented for descriptive purposes only; Table S1: Pre-specified parsimonious Cox proportional hazards model for biochemical recurrence-free survival.
