# Supplementary material for: Neoadjuvant ADT for Asian Patients Undergoing Robotic Radical Prostatectomy Is the Conversation Over?—A Propensity-Matched Comparison
Source: Cancers (Basel). 2026 Feb 18;18(4):661. doi: 10.3390/cancers18040661 (PMC12940029; doi:10.3390/cancers18040661)
Supplement: Supplementary file 1 [file cancers-18-00661-s001.zip › cancers-4136877-supplementary.pdf]

cancers

MDPI

Supplementary Materials: Neoadjuvant ADT for Patients undergoing Robotic Radical Prostatectomy, is the Conversation over?—A Propensity Matched Comparison

John Joson Ng, Sean Lim, Alvin Lee, Yu Guang Tan, Kae Jack Tay, Henry Ho, John Yuen and Chen Kenneth

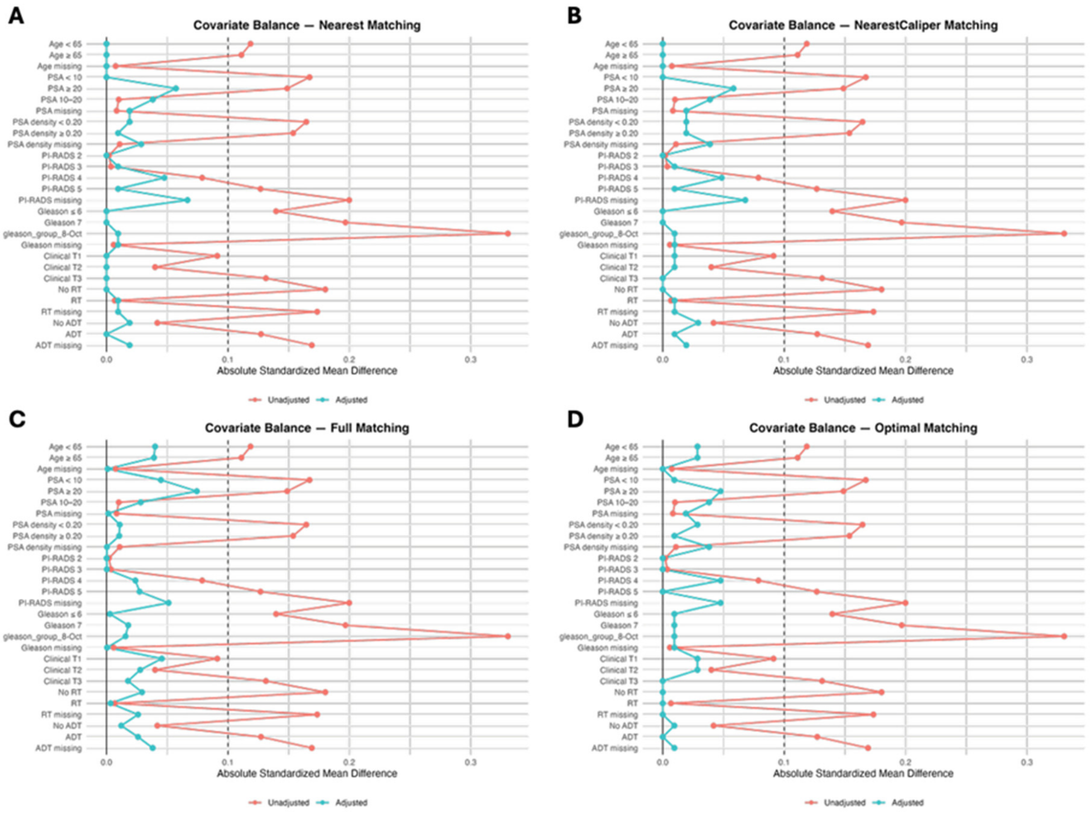

Figure S1. Covariate Balance Across Matching Algorithms: Absolute Standardized Mean Differences Before and After Matching. Covariate balance before and after propensity-score matching using four different algorithms. Each panel plots the absolute standardized mean differences (ASMD) for all baseline covariates (age groups, PSA categories and density, PI-RADS, Gleason score, clinical T stage, radiotherapy [RT] status, and ADT status), with red dots and lines showing the unadjusted ASMDs and blue dots and lines showing the ASMDs after matching. The vertical dashed line at 0.10 indicates the commonly accepted threshold for adequate balance. (**A**) Nearest-neighbor matching (without caliper); (**B**) Nearest-neighbor matching with caliper (0.2); (**C**) Full matching; (**D**) Optimal matching.

Cancers **2026**, *18*, 661 https://doi.org/10.3390/cancers18040661

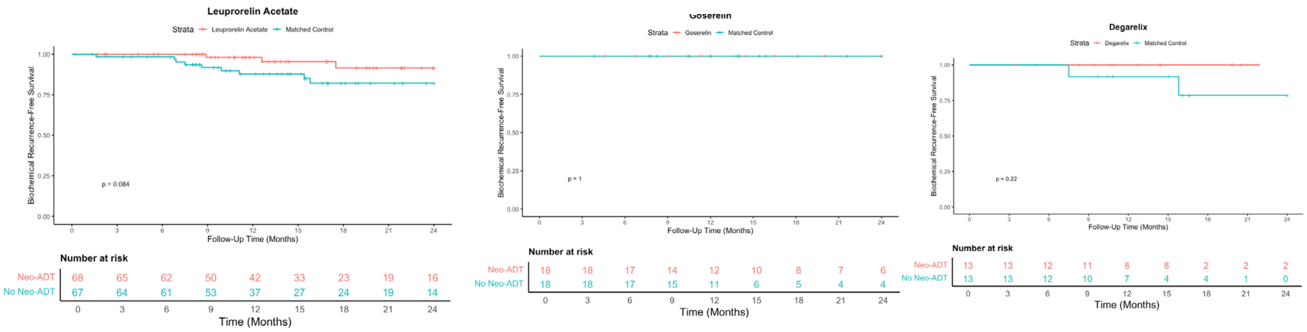

(a) (b) (c)

Figure S2. Agent-specific Kaplan–Meier curves for biochemical recurrence–free survival comparing neoadjuvant androgen deprivation therapy (ADT) recipients with their matched control cohorts. (**a**) Leuprorelin acetate (**b**) Goserelin (**c**) Degarelix. Owing to the limited number of biochemical recurrence events within individual ADT agent subgroups, these analyses are underpowered for formal statistical comparison and are presented for descriptive purposes only.

Table S1. Pre-specified parsimonious Cox proportional hazards model for biochemical recurrence–free survival.

Variable	Hazard Ratio	95% CI	*p*-Value
Neoadjuvant ADT (yes vs no)	0.28	0.09–0.80	0.018
Age ≥65 years	0.71	0.28–1.78	0.46
PSA 10–20 ng/mL	0.57	0.15–2.12	0.4
PSA ≥20 ng/mL	1.6	0.57–4.44	0.37
Clinical T2	2	0.61–6.50	0.25
Clinical T3	2.41	0.83–7.02	0.11
PSA group (missing)	10.67	1.11–102.90	0.041
Gleason group 7	—	—	—
Gleason group ≥8	—	—	—
Gleason group (missing)	—	—	—

Hazard ratios (HRs) and 95% confidence intervals (CIs) were estimated using a Cox proportional hazards model adjusting for age group, PSA group, Gleason group, and clinical T stage. This model was specified a priori as a sensitivity analysis for the primary endpoint. Due to the limited number of biochemical recurrence events, some covariate estimates were unstable or not estimable and should be interpreted with caution. Interpretation focuses on the treatment effect of neoadjuvant androgen deprivation therapy.

https://doi.org/10.3390/cancers18040661
